# Human Osteoblast-Conditioned Media Can Influence *Staphylococcus aureus* Biofilm Formation

**DOI:** 10.3390/ijms232214393

**Published:** 2022-11-19

**Authors:** Fabien Lamret, Jennifer Varin-Simon, Mélodie Six, Léa Thoraval, Julie Chevrier, Cloé Adam, Christine Guillaume, Frédéric Velard, Sophie C. Gangloff, Fany Reffuveille

**Affiliations:** 1 Biomatériaux et Inflammation en Site Osseux, BIOS EA 4691, SFR Cap Santé, Université de Reims Champagne-Ardenne, 51097 Reims, France; 2 UFR Pharmacie, Service de Microbiologie, Université de Reims Champagne-Ardenne, 51097 Reims, France

**Keywords:** MSSA, MRSA, biofilms, prosthetic joint infection, osteoblast secretion

## Abstract

Osteoblasts are bone-forming and highly active cells participating in bone homeostasis. In the case of osteomyelitis and more specifically prosthetic joint infections (PJI) for which *Staphylococcus aureus* (*S. aureus*) is mainly involved, the interaction between osteoblasts and *S. aureus* results in impaired bone homeostasis. If, so far, most of the studies of osteoblasts and *S. aureus* interactions were focused on osteoblast response following direct interactions with co-culture and/or internalization models, less is known about the effect of osteoblast factors on *S. aureus* biofilm formation. In the present study, we investigated the effect of human osteoblast culture supernatant on methicillin sensitive *S. aureus* (MSSA) SH1000 and methicillin resistant *S. aureus* (MRSA) USA300. Firstly, Saos-2 cell line was incubated with either medium containing TNF-α to mimic the inflammatory periprosthetic environment or with regular medium. Biofilm biomass was slightly increased for both strains in the presence of culture supernatant collected from Saos-2 cells, stimulated or not with TNF-α. In such conditions, SH1000 was able to develop microcolonies, suggesting a rearrangement in biofilm organization. However, the biofilm matrix and regulation of genes dedicated to biofilm formation were not substantially changed. Secondly, culture supernatant obtained from primary osteoblast culture induced varied response from SH1000 strain depending on the different donors tested, whereas USA300 was only slightly affected. This suggested that the sensitivity to bone cell secretions is strain dependent. Our results have shown the impact of osteoblast secretions on bacteria and further identification of involved factors will help to manage PJI.

## 1. Introduction

During infections, complex interkingdom communication occurs between pathogenic bacteria and host cells, and not only with immune cells [1]. Deciphering pathogens and host interactions is crucial to depict the infectious environment and find more adapted therapeutic strategies. Indeed, bacteria can trigger host cells production of inflammatory molecules but these cells could in counterpart increase bacterial virulence characteristics [1,2,3,4,5].

Prosthetic joint infections (PJI) are classically defined as a colonization of the implant and adjacent tissue. However, continued research is warranted to characterize the periprosthetic microenvironment. Indeed, the infectious microenvironment influence on antibacterial therapies is a growing concern [5]. Biofilm colonization is often related to these infections and linked to persistence and chronicity [6,7,8]. *Staphylococcus aureus* (*S. aureus*) is the most involved bacterium in PJI and more globally in osteomyelitis [9,10].

The interaction between planktonic *S. aureus* and bone cells such as bone-forming osteoblast has been widely studied [1]. Thus, *S. aureus* possess an arsenal of toxins and immunomodulatory molecules which can adversely affect osteoblasts by inducing apoptosis or lead to a reduction of their proliferation and mineralization capacity [1,11]. Furthermore, *S. aureus* is able to invade osteoblasts and *S. aureus* toxin α-type phenol soluble modulins promote the intracellular survival of the bacteria [12,13]. On the other hand, osteoblasts could play a role in inflammatory immune responses through, for instance, the production of defensin in response to the bacterial presence [1,14,15].

*S. aureus* and osteoblast interaction studies are mainly focused on direct contact between cells and bacteria (co-culture models, internalization) but less is known on the effect of unchallenged osteoblast secretions on *S. aureus* biofilm formation [1]. Actually, osteoblasts unstimulated by the presence of bacteria better mimic the condition encountered just after joint arthroplasty, i.e., the environment that bacteria will encounter at the initiation of infection. Furthermore, an inflammatory environment challenges host cells. Prosthesis itself and particles arising from prosthesis such as particulate titanium, cobalt-chromium, and stainless steel as well as the polymethylmethacrylate (used as bone cement) particles lead to the production by host cells of tumor necrosis factor-alpha (TNF-α), a proinflammatory cytokine [16,17]. Thus, osteoblasts (stimulated or not by a biomaterial) could influence bacterial behavior and biofilm formation during an infection. Osteoblasts-secreted factors could act as anti-biofilm through the production of anti-microbial peptides (AMPs) for example or lead to an induction of biofilm formation through a stress response [15,18].

In the present study, we investigated *S. aureus* biofilm formation in response to the presence of osteoblast-conditioned media harvested from osteoblast-like cells (Saos-2) and primary osteoblasts cultures to evaluate the pro- or anti-biofilm effect of osteoblast-produced molecules.

## 2. Results

### 2.1. Effect of Saos-2 Culture Supernatant, Stimulated or Not with TNF-α, on Planktonic Growth and Biofilm Formation

In order to investigate the effect of Saos-2 secretions on planktonic growth and biofilm formation of *S. aureus*, Saos-2 cells were cultivated to harvest the supernatants. Cells were cultivated for three days without or with TNF-α to mimic the inflammatory environment following arthroplasty. The day of the experiment, the bacterial culture medium (minimal medium) was added to supernatants (50:50) to avoid stresses induced by nutrient depletion due to Saos-2 cell consumption, as described in a previous study [2]. The control condition corresponded to the addition of fresh culture media for eukaryotes to minimal medium (50:50). This led to four conditions: namely “Control media”, “SN 50”, “Control media + TNF-α”, and “SN 50 + TNF-α”. Bacteria were incubated in these conditions for 24 h under hypoxic condition to get closer to bone environment [2,4].

**Figure 1:**
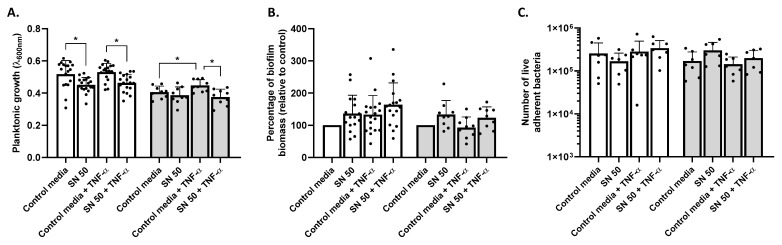
Impact of Saos-2 culture supernatant on *S. aureus* planktonic growth and biofilm biomass. Results represent planktonic growth (**A**), percentage of biofilm biomass relative to control media (**B**), and number of adherent bacteria (**C**) for SH1000 (white histogram) and USA300 (grey histogram). Control media (50% [DMEM + 10% FBS] and 50% MM), SN 50 (50% Saos-2 culture supernatant and 50% MM), Control media + TNF-α (50% [DMEM + 10% FBS + 20 ng/mL TNF-α] and 50% MM), or SN 50 + TNF-α (50% [Saos-2 challenged with TNF-α culture supernatant + 20 ng/mL] and 50% MM). *n* = 7 to 18. * = *p* < 0.05.

Our first approach was to measure planktonic growth and biofilm biomass under these conditions to evaluate the impact of Saos-2 secretions. A significant decrease in planktonic growth was noticed for SH1000 in response to Saos-2 culture supernatant, challenged or not with TNF-α (12.7 and 12.9%, respectively, *p* < 0.05) (Figure 1A). For USA300, only culture supernatants from TNF-α-challenged cells significantly decreased the planktonic growth (16.2%, *p* < 0.05) compared to control media supplemented with TNF-α. The control media containing TNF-α induced a significant increase in planktonic growth for USA300 (10.2%, *p* < 0.05) compared to the control media without TNF-α. Regarding relative biofilm biomass, no significant changes were noticed (Figure 1B). Raw data of biofilm biomass measurement are available in Figure S1. However, compared to control media, supernatants from Saos-2 tended to increased biofilm biomass by 10.1% for SH1000 and 10.5% for USA300. Non-significant increased biofilm biomass was further noticed with culture supernatants from Saos-2 challenged with TNF-α for SH1000 (22.9%) and USA300 (24.5%).

To complete our approach on biofilm evaluation, we proceeded with the numeration of live and adherent bacteria contained within biofilms (Figure 1C). SH1000 and USA300 did not display significant changes in the number of bacteria compared to control media.

### 2.2. Effect of Saos-2 Culture Supernatant, Stimulated or Not with TNF-α, on Biofilm Structure

**Figure 2:**
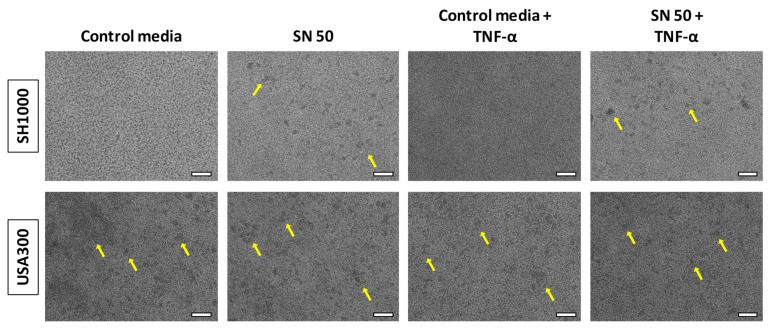
Impact of Saos-2 culture supernatant on *S. aureus* biofilm organization. Yellow arrows indicate aggregates. Control media (50% [DMEM + 10% FBS] and 50% MM), SN 50 (50% Saos-2 culture supernatant and 50% MM), Control media + TNF-α (50% [DMEM + 10% FBS + 20 ng/mL TNF-α] and 50% MM), or SN 50 + TNF-α (50% [Saos-2 challenged with TNF-α culture supernatant + 20 ng/mL] and 50% MM). One representative image of each technical duplicate was acquired for each condition of each batch (for one biological replicate). The scale bars indicate 50 µm.

As revealed by light microscopy, SH1000 tended to form more aggregates that could be associated to biofilm microcolonies for SN 50 + TNF-α conditions, especially for two batches (Figure 2). The third batch induced bigger aggregates and no difference between conditions (Figure S2). USA300 seemed to form aggregates in every condition.

**Figure 3:**
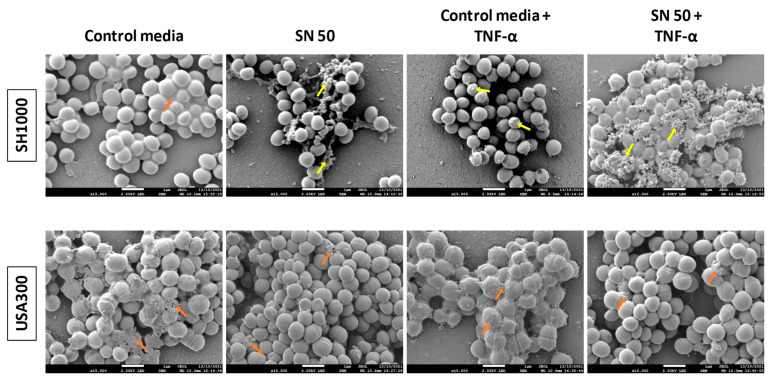
Impact of Saos-2 culture supernatant on *S. aureus* biofilm shape and matrix. One representative image for two batches is shown. Control media (50% [DMEM + 10% FBS] and 50% MM), SN 50 (50% Saos-2 culture supernatant and 50% MM), Control media + TNF-α (50% [DMEM + 10% FBS + 20 ng/mL TNF-α] and 50% MM), or SN 50 + TNF-α (50% [Saos-2 challenged with TNF-α culture supernatant + 20 ng/mL] and 50% MM). Orange arrows indicate bacterial matrix. Yellow arrows indicate microvesicles-like structure. One coverslip was imaged for two different batches of Saos-2 culture supernatant and the corresponding control media. The scale bars indicate 1 µm.

SEM examination of biofilms has shown that each condition induced matrix production (Figure 3). USA300 biofilm matrix seemed homogeneous in every condition. Regarding the batch shown in Figure 3, SH1000 matrix seemed homogeneous with control media without TNF-α (orange arrow), whereas microvesicle-like structures were present in the three other conditions (yellow arrows). From a general point of view, we noticed more matrix structures in SH1000 biofilm in the presence of SN 50 and even more with SN 50 + TNF-α contrary to USA300 biofilm.

### 2.3. Effect of Saos-2 Culture Supernatant, Stimulated or Not with TNF-α, on Composition of Biofilm Matrix

**Figure 4:**
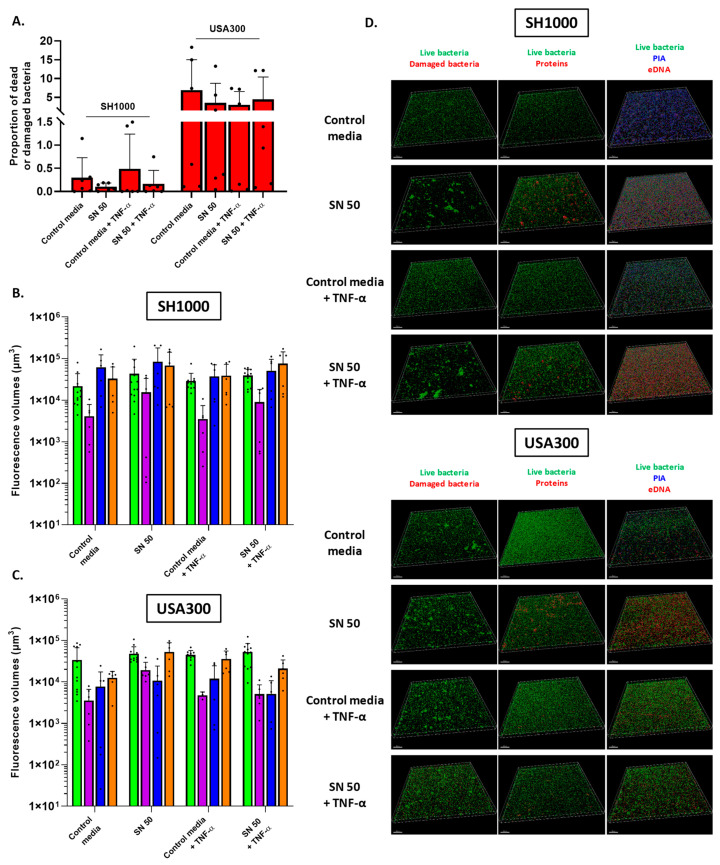
Impact of Saos-2 culture supernatant on proportion of dead cells and biofilm matrix components of *S. aureus*. (**A**) Proportion of dead or damaged bacteria; volume measurement of SYTO^TM^ 9 and propidium iodide were acquired by CLSM. (**B**,**C**) Volume measurement of SYTO^TM^ 9 (green histograms, live bacteria), SYPRO^®^ Ruby (purple, protein), WGA (blue, PIA), and TOTO^TM^-3 (orange, extracellular DNA) acquired by CLSM for SH1000 and USA300 biofilms, respectively. (**D**) Representative reconstructions of *S. aureus* biofilms are shown. Above each column is indicated the labelled component and the corresponding colour. The scale bars indicate 50 µm. Control media (50% [DMEM + 10% FBS] and 50% MM), SN 50 (50% Saos-2 culture supernatant and 50% MM), Control media + TNF-α (50% [DMEM + 10% FBS + 20 ng/mL TNF-α] and 50% MM), or SN 50 + TNF-α (50% [Saos-2 challenged with TNF-α culture supernatant + 20 ng/mL] and 50% MM). *n* = 2 with three acquisitions per coverslip; dots represent technical replicates for this figure.

To find out the nature of the observed matrix and trend of changes, we performed fluorescent labelling of the main components of the matrix of *S. aureus* biofilm, as well as the labelling of dead or damaged bacteria. Regarding this last point, SH1000 biofilms contained less than 0.5% of dead or damaged bacteria. Three to six% of dead or damaged bacteria were composed in USA300 biofilms without significant changes between conditions (Figure 4A). SH1000 presented bacteria-labelled volumes of 2.2 × 10^4^ µm^3^ and 2.9 × 10^4^ µm^3^ for control media and control media supplemented with TNF-α, respectively (Figure 4B). Saos-2 cells supernatants tended to increase the measured bacteria-labelled volumes, which reached 4.3 × 10^4^ µm^3^ (with high standard deviation) and 3.9 × 10^4^ µm^3^ for Saos-2 cells challenged with TNF-α. PIA and eDNA were the main components of the matrix of SH1000 biofilms. The biofilms of USA300 developed with control media was constituted of 3.3 × 10^4^ µm^3^ of bacteria-labelled volume with high standard deviation (Figure 4C). The three other conditions increased the bacteria-labelled volumes, which reached 4.4 × 10^4^ to 5.2 × 10^4^ µm^3^. The eDNA was the main matrix component quantified. Proteins were in the minority for both strains and each condition (Figure 4B,C). Representative images are shown in Figure 4D.

### 2.4. Effect of Saos-2 Culture Supernatant, Stimulated or Not with TNF-α on Gene Expression Involved in Biofilm Formation

**Figure 5:**
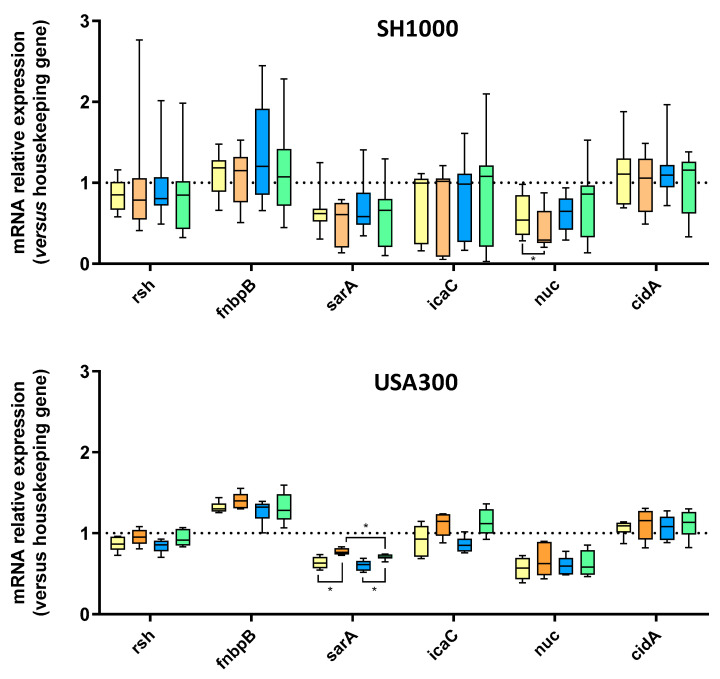
Influence of Saos-2 culture supernatant on biofilm-embedded *S. aureus* gene regulation. Results represent relative mRNA expressions of bacteria within biofilms for SH1000 and USA300. Control media (Yellow histograms = 50% [DMEM + 10% FBS] and 50% MM), SN 50 (Orange histograms = 50% Saos-2 culture supernatant and 50% MM), Control media + TNF-α (Blue histograms = 50% [DMEM + 10% FBS + 20 ng/mL TNF-α] and 50% MM), or SN 50 + TNF-α (Green histograms = 50% [Saos-2 challenged with TNF-α culture supernatant + 20 ng/mL] and 50% MM). Experiments were performed nine (SH1000) and six (USA300) independent times. *n* = 6 to 9, * *p* < 0.05.

Finally, we investigated regulation of genes involved in biofilm formation using RT-qPCR. Stress-responses-related gene *rsh*, adhesion-related gene *fnbpB*, and biofilm formation/matrix production-related genes (*sarA*, *icaC*, *nuc*, and *cidA*) were thus studied [19,20,21,22,23,24]. SH1000 displayed highly dispersed results (Figure 5). Among the tested genes, only nuc was significantly downregulated by 46% (from 0.54, *p* < 0.05) for Saos-2 supernatant compared to control media. Regarding USA300, only *sarA* regulation was significantly changed: incubation with Saos-2 culture supernatants increased *sarA* expression by 1.21-fold (from 0.63, *p* < 0.01) compared to control media (Figure 5). In the same way, incubation with TNF-α-challenged Saos-2 culture supernatants increased *sarA* regulation by 1.15-fold (from 0.61, *p* < 0.01) compared to control media supplemented with TNF-α. *IcaC* was downregulated in the presence of control media (to 0.93) and control media + TNF-α (to 0.85) and upregulated in the presence of SN 50 (to 1.15) and SN 50 + TNF-α (to 1.12).

### 2.5. Effect of Primary Osteoblast Culture Supernatant on Biofilm Formation

**Figure 6:**
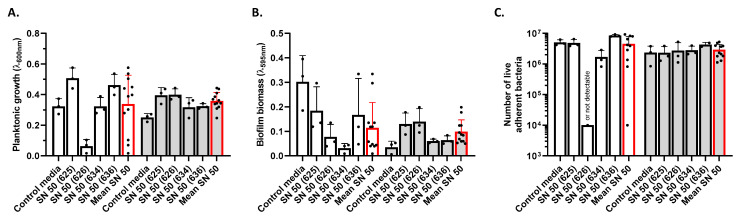
Impact of primary osteoblast culture supernatant on *S. aureus* planktonic growth and biofilm biomass. Results represent planktonic growth (**A**), biofilm biomass (**B**), and number of live adherent bacteria (**C**) for SH1000 (white histogram) and USA300 (grey histogram). Control media (50% [DMEM + 10% FBS] and 50% MM), SN 50 (625, 626, 634, and 636) (50% primary human osteoblast culture supernatant and 50% MM), or All SN 50 (addition of results of SN 50 (625, 626, 634, and 636)). *n* = 2 to 3.

We next investigated the biofilm phenotype influenced by human primary osteoblast culture supernatants. For SH1000, the results were heterogeneous. Both SN 50 (625) and (636) tended to increase the planktonic growth of this strain; SN 50 (634) had no impact whereas SN 50 (626) greatly decreased the planktonic growth (Figure 6A). However, each supernatant from different cell donors decreased biofilm biomass (Figure 6B). USA300 was moderately impacted by these supernatants: all donors tended to increase planktonic growth (43.2% on average) (Figure 6A) and biofilm biomass (180% on average) (Figure 6B). However, two donors tended to increase more substantially the biofilm biomass for this strain: SN 50 (625) and SN 50 (626).

For SH1000, donor SN 50 (625) did not change the number of live adherent bacteria, whereas SN 50 (634) tended to reduce the bacterial burden (by 66%) and SN 50 (626) reduced it to the point that no CFU was detected (Figure 6C). Meanwhile, SN 50 (636) increased the number of live adherent bacteria (1.68-fold). Regarding USA300, the number of live adherent bacteria tended to increase (1.25-fold on average), although this increase was mainly due to SN 50 (636).

**Figure 7:**
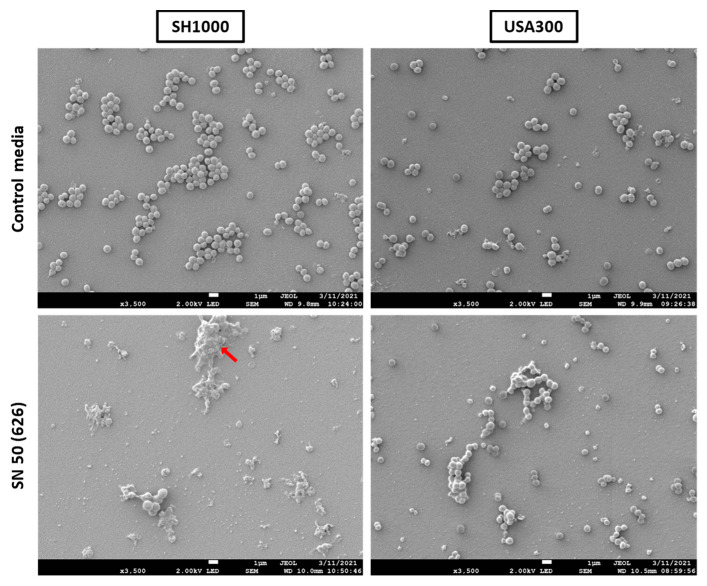
Impact of primary osteoblast culture supernatant on *S. aureus* biofilm shape and matrix. Control media (50% [DMEM + 10% FBS] and 50% MM), SN 50 (626) (50% primary human osteoblast culture supernatant and 50% MM), or All SN 50 (addition of results of SN 50 (625, 626, 634 and 636)). Red arrow indicates agglomerated dead bacteria. Two coverslips were imaged for each donor of primary osteoblast culture supernatant and the corresponding control media. The scale bars indicate 1 µm.

Examination of biofilms with the aforementioned conditions did not reveal matrix structures for both SH1000 and USA300 biofilms, except that agglomerated structures were observed for SH1000 grown with SN 50 (626) (Figure 7). We assimilated these structures with dead bacteria. No differences in biofilm structures were observed between conditions for SH1000 and USA300 according to the different donors (except for SH1000 and SN 50 (626)). More images are available in Figure S3.

## 3. Discussion

We previously investigated MSSA CIP 53.154 biofilm formation under aerobic condition and showed that culture supernatants from Saos-2 cells stimulated with TNF-α increased bacterial adhesion without impacting the overall biomass [2]. In the present study, we investigated biofilm formation by MSSA SH1000 and MRSA USA300 in the same media containing Saos-2 cell secretion, although bacteria were incubated in hypoxia to get closer to the oxygen concentration within the periprosthetic environment [25]. As previously conducted, minimal medium was added to Saos-2 culture supernatants to overcome nutrient starvation due to eukaryotic cell consumption, which is too severe [2]. In doing so, Saos-2 secreted factors were diluted, possibly attenuating the observed bacterial responses.

Despite the fact that culture supernatants of Saos-2 cells stimulated or not with TNF-α were supplemented with MM, we observed a weak bacteria planktonic growth deficit. This phenomenon was not observed previously in aerobic condition of CIP 53.154 *S. aureus* strains [2]. Studies have shown that bacteria-challenged osteoblasts can produce antimicrobial peptides, inflammatory cytokines, and chemokines [1,26,27,28]. Here, we speculated that constitutive production of some of these factors could affect bacterial growth. Interestingly, control media containing TNF-α slightly increased the planktonic growth of USA300, suggesting an effect of this cytokine but without affecting biofilm. TNF-α has already been described to modify *S. aureus* bacterial growth [29].

As far as biofilms are concerned, both SH1000 and USA300 biofilm biomass were slightly modified in contact of diluted Saos-2 culture supernatant, with a tendency to be increased, suggesting that Saos-2 produced factors that influenced biofilm formation. These data echo findings from our previous study [2]. However, modification of biofilm behavior was sometimes variable and seemed to be batch dependent.

A strong induction of biofilm formation could lead to thick biofilms, which is more difficult to eradicate. A non-significant increase of biofilm biomass correlated with the number of live adherent bacteria were induced by both culture supernatants for USA300. No changes in biofilm aggregation were observed through light microscopy. However, light microscopy revealed microcolonies formation for SH1000 strain in the presence of the two different types of Saos-2 culture supernatants and not encountered for control medium. Live adherent bacterial burdens were not correlated with biofilm biomass changes for SH1000 strain, revealing a potential difference in matrix production. Taken together, these data confirmed that culture supernatants of Saos-2 influenced *S. aureus* biofilm formation, in a strain-dependent way: a possible modification in matrix production and biofilm structure for MSSA SH1000 and increase in adhered bacteria for MRSA USA300 leading to investigate the biofilm matrix and non-growing bacteria (dead or damaged bacteria).

To investigate biofilm matrix, an important characteristic of biofilms, we next used microscopy. SEM revealed that USA300 always exhibited a same-aspect matrix, which seemed “fibrous”. CLSM approach has shown that this matrix was mainly composed of eDNA, suggesting that the “fibrous” aspect corresponded to a predominance of eDNA. Saos-2 culture supernatant increased slightly the proportion of proteins and more importantly the proportion of eDNA. TNF-α alone (control media with TNF-α) also increased this eDNA proportion, which was reduced by the influence of culture supernatants of Saos-2 challenged with TNF-α. However, we did not observe major modifications in matrix composition. SH1000 produced aggregates as observed by light microscopy for two batches on three and a matrix of different nature according to the condition, as observed on SEM images. With control media, the matrix of this strain seemed “slimy”, and labelling of the main matrix components suggested that this was due to a predominance of PIA. Saos-2 culture supernatant with or without TNF-α challenge induced a small change in the matrix, which looked like microvesicles. At the same time, the Saos-2 culture supernatant increased live-bacteria volume, with no important changes in the proportion of the different matrix components, except a slight increase in proteins and eDNA proportion. We noticed that TNF-α alone (control media with TNF-α) reduced the amount of PIA, compared to the control media. The predominance of PIA for MSSA and eDNA for MRSA in their respective matrix is concordant with the literature [30].

In order to detect potential gene regulation changes in response to Saos-2 culture supernatant, different genes involved in biofilm formation were investigated by RT-qPCR. Results obtained for SH1000 strain were highly dispersed, which revealed heterogeneous responses among SH1000 bacteria to Saos-2 supernatants. However, *nuc* was downregulated in the presence of Saos-2 supernatants and its regulation was disturbed with TNF-α-challenged Saos-2 culture supernatant. This gene is involved in eDNA cleavage and inhibition of biofilm initiation [23]. The decrease in relative *nuc* expression was correlated with the increase in eDNA, revealed by CSLM quantifications. Regarding USA300, *sarA* relative expression was increased by both culture supernatants and *icaC* was upregulated in response to Saos-2 culture supernatants whereas this gene was downregulated with control media (with or without TNF-α), but surprisingly this was not correlated with PIA production as shown by CLSM quantification. Thus, SH1000 strain and USA300 seemed to be influenced by the presence of Saos-2 secretome but in a different way: heterogeneous response affecting various genes for the first one and homogeneous and specific answer with biofilm-involved gene regulation for the second one.

To conclude on this first approach, Saos-2 secretion affected *S. aureus* biofilm formation but in a strain-dependent manner. We observed a heterogeneous response for MSSA SH1000 biofilm, with a modification of structure in the presence of the supernatants in a batch-dependent manner. Concerning MRSA USA300, we only observed a slight increase in adhered bacteria, supposedly due to *sarA* gene regulation, involved in bacterial adhesion without influence on biofilm characteristics [31].

To find out whether the observed changes in biofilm phenotype were consistent with more representative conditions, primary osteoblast culture supernatants were harvested to investigate their influence on *S. aureus*. Globally, the culture supernatants of primary osteoblast tended to increase USA300 planktonic growth, biofilm biomass, and live adherent bacterial burden. Interestingly, SH1000 was subjected to important variation of responses to the different donors. While each donor tended to decrease the biofilm biomass, the planktonic growth and the live adherent bacterial burden were sometimes increased. Surprisingly, the donor “626” clearly reduced the biofilm formation of SH1000, linked with a decreased planktonic growth, whereas USA300 was not impacted. We conclude that the influence of Saos-2 culture supernatants is strain and donor dependent. These media could contain pro- and/or anti-biofilm factors.

We investigated the cytokine composition of osteoblast culture supernatant (Table S1). ELISA assays confirmed that Saos-2 cells constitutively secreted MCP-1 and responded to TNF-α stimulation by producing more IL-6 and IL-8 and MCP-1. Same cytokines were quantified in primary osteoblast culture. No TNF-α was detected, IL-8 production was heterogeneous, a lot of MCP-1 was quantified, and more IL-6 was produced by primary osteoblasts. Further investigations are needed to understand the difference in cytokines production and the consequences on biofilm formation.

*S. aureus* SH1000 planktonic growth and biofilm formation were clearly affected in a different way according to osteoblast donors. Thus, identification of the osteoblast factors affecting biofilm development in positive or negative ways will lead to new therapeutic strategies targeting immunomodulatory responses. Our results suggested that SH1000 strain was more sensitive to the primary osteoblast factors. Even if this last point needs to be confirmed with other strains and clinical isolates, studies have already proven that different *S. aureus* strains did not respond in the same way [32,33]. In this study, USA300 biofilm formation was similar in the presence of Saos-2 or primary cell secretions, with a slight increase in adhered bacteria. SH1000 biofilm is heterogeneous, donor dependent, and sensitive to possible antimicrobial osteoblast products. USA300, as a MRSA strain, is also less susceptible to β-defensin, which could partially explain the differential bacterial response displayed by SH1000 and USA300 strains in the presence of primary osteoblast supernatants [34]. However, the observed differences could be due to genetic variation (e.g., adhesin genes more prevalent in MRSA) of the strains more than their antibiotic sensitivity characteristic [33]. In general, MRSA isolates carried more virulence genes than MSSA isolates but without influencing the outcome of infection [33].

In perspective, other investigations are needed to identify osteoblast secretome factors affecting biofilm formation. We already treated Saos-2 culture supernatants with proteinase K in order to eliminate proteins and the observed increases in biofilm formation disappeared for SN 50 + TNF-α and confirmed the proteinaceous origin of the observed results (Figure S4).

## 4. Materials and Methods

### 4.1. Osteoblast Culture and Supernatant Gathering

The Saos-2 cell line (ATCC^®^ HTB-85^TM^) was cultured at 37 °C in a 5% CO_2_ humidified atmosphere in Dulbecco’s modified Eagle’s medium (DMEM, Gibco, Paisley, UK) supplemented with 10% fetal bovine serum (FBS, Dutscher, Bernolsheim, France) and 1% antibiotic solution PenStrep^®^ (Gibco, Paisley, UK) considered as standard medium (SM). Saos-2 cells were grown to 60–80% confluence in SM and then rinsed with sterile Dulbecco’s Phosphate Buffered Saline (DPBS, Gibco, Paisley, UK) to eliminate antibiotics. Saos-2 cells were further incubated with DMEM and 10% FBS, without antibiotics, supplemented or not with recombinant human tumor necrosis factor α (TNF-α) at 20 ng/mL (R&D Systems, Minneapolis, MN, USA) in 75 cm^2^ flasks. After 72 h of incubation, collected culture supernatants of Saos-2 cells and control media were stored at −80 °C. Three different batches of Saos-2 culture supernatants were used for each assay, except for confocal laser scanning microscopy (CLSM) and scanning electron microscopy (SEM), as described below.

Regarding primary human osteoblasts, femoral bone explant samples were obtained from patients having undergone total knee arthroplasty (Conservation d’Élément du Corps Humain Authorization DC-2014-2262 given by the French Ministry of Higher Education and Research and Innovation). After sampling, primary human osteoblasts from four different donors named “625”, “626”, “634”, and “636” were obtained by migration from cancellous bone explants placed in culture, as described previously [35]. Fragments were cut into small pieces, washed in DPBS four times for 10 min, digested in a solution of 0.5% trypsin-EDTA (Gibco, Paisley, UK), and then in 0.14% (*w*/*v*) type II collagenase three times (30 min each time) (Sigma Aldrich, St. Louis, MO, USA). The fragments obtained were placed in 25 cm² culture flasks containing DMEM supplemented with 20% FBS and 1% PenStrep^®^ and incubated at 37 °C in a 5% CO_2_ humidified atmosphere to allow cell migration from explants. When cells reached confluence, they were amplified in 75 cm² culture flasks with 10% FBS containing medium. The amplification step was repeated two times. In all experiments, cell culture supernatants (without antibiotics) were collected at the third passage. As for Saos-2 culture supernatants, primary human osteoblast culture supernatants and control media were stored at −80 °C.

### 4.2. Bacterial Strains

Two *S. aureus* strains were used in this study: SH1000 and USA300. SH1000 is a MSSA originated from 8325-4 strain with *rsbU* gene repaired [36]. USA300 strain, meanwhile, emerged first as community-associated MRSA in the United States in the late 1990s and became endemic pathogens worldwide. This strain is known to be implicated in osteomyelitis and was first reported in the United States as a cause of skin and soft issue infection (Tenover and Goering, 2009). To prepare the inoculum, −20 °C glycerol stocks were steaked out on tryptic soy agar plates. Overnight cultures were performed by transferring one colony of a strain into 3 mL nutritive broth (Bio-Rad, Marnes-la-Coquette, France) for 18 h at 37 °C.

### 4.3. S. aureus Incubation with Osteoblast Culture Supernatant

For all experiments, the absorbance of *S. aureus* overnight cultures was measured at 600 nm. Overnight cultures were then diluted in minimal medium (MM) to obtain an expected absorbance of 0.02, except for real-time quantitative PCR (RT-qPCR): overnight cultures were diluted in MM to obtain an expected absorbance of 0.2. The MM consisted of 62 mM potassium phosphate buffer, pH 7.0, 7 mM (NH^4^)^2^SO^4^, 2 mM MgSO^4^, 10 µM FeSO^4^), 0.4% (*w*/*v*) glucose, and 0.5% (*w*/*v*) casamino acids.

MM containing bacteria was then mixed with Saos-2 or primary osteoblast culture supernatants or control media (50:50) and consisted of the bacterial suspension (BS) inoculated in microplates and incubated at 37 °C for 24 h. We included four conditions for the Saos-2 culture supernatant study: “Control media” (50% of fresh [DMEM + 10% FBS] and 50% MM), “SN 50” (50% Saos-2 culture supernatants and 50% MM), “Control media + TNF-α” (50% of fresh [DMEM + 10% FBS + 20 ng/mL TNF-α] and 50% MM), and “SN 50 + TNF-α” (50% [Saos-2 culture supernatants + 20 ng/mL TNF-α] and 50% MM). Regarding primary osteoblast culture supernatants, the control media was composed of 50% of MM and 50% of fresh media, and the other conditions consisted of 50% of MM and 50% of the culture supernatants obtained from the four donors which were named “SN 50 (625); (626); (634) or (636)”. All biofilm assays were performed under hypoxic condition using the GenBag system (Biomérieux, Marcy L’Étoile, France). The number of biological repeats (different bacteria overnight culture) and technical repeats (different wells for the same bacteria overnight culture) were dependent of the technics used for the experiment and are indicated in the corresponding technics parts in this Methods Section.

### 4.4. Crystal Violet Staining

For each condition, BS were distributed in 48 well plates (500 µL/well) and incubated at 37 °C for 24 h. The planktonic growth was evaluated by measuring the absorbance at 600 nm. The wells were gently washed three times with water, and 500 µL of 0.2% crystal violet was added to each well. Plates were incubated for 20 min in the dark at room temperature. The wells were washed three times with water, and 500 µL of 95% ethanol was added to each well. The absorbance at 595 nm was measured to evaluate the amount of biofilm biomass, which is proportional to the absorbance value. Regarding experiments involving Saos-2 culture supernatants, nine to eighteen biological replicates (each batch representing a third of these replicates) were used, each including three technical replicates. Regarding experiments involving primary osteoblast culture supernatants, three biological replicates by donors were used, each including three technical replicates.

### 4.5. Counting Method

To assess the number of live adherent bacteria, BS were distributed in 24 well plates (500 µL per well) containing Thermanox^TM^ coverslips (Thermo Fisher Scientific, Waltham, MA, USA). After 24 h of incubation at 37 °C, the coverslips were washed with MM and transferred to a 15 mL tube containing 2 mL of MM. Bacteria attached to coverslips were then detached by placing the tube in ultrasonic bath (40 kHz) for 5 min. A volume of 100 µL from serial dilutions was plated on nutrient agar plates. Regarding experiments involving Saos-2 culture supernatants, at least two biological replicates per batch were performed, for three different batches, each including two technical replicates. Regarding experiments involving primary osteoblast culture supernatants, at least two biological replicates by donors were performed, each including three technical replicates.

### 4.6. Light Microscopy

For each condition, BS were distributed into a 24 well plate (500 µL per well) and incubated for 24 h at 37 °C. Wells were washed twice with distilled water before imaging. Light microscopy images of the well’s bottom were performed using a Zeiss Axiovert 200 M inverted microscope (Zeiss, Oberkochen, Germany) using 20× objective (and coupled AxioVision^TM^ v.8 software). For three batches of Saos-2 culture supernatant, one representative image of each technical replicate (two in total) of one biological experiment by batch was acquired.

### 4.7. Scanning Electron Microscopy

For each condition, BS were distributed onto a Thermanox^TM^ coverslip (cell culture treated side up) placed at the bottom of a 24 well plate (500 µL per well). After 24 h of incubation at 37 °C, the coverslips were washed twice in phosphate-buffered saline (PBS), and then fixed in 2.5% (*w*/*v*) glutaraldehyde (Sigma-Aldrich, St. Louis, MO, USA) at room temperature for 1 h. After two distilled water rinses, biofilms were dehydrated in graded ethanol solutions (50, 70, 90, and 100% twice) for 10 min. Biofilms were finally desiccated in a drop of hexamethyldisilazane (Sigma-Aldrich, St. Louis, MO, USA). After air drying at room temperature, samples were sputtered with a thin gold–palladium film using a JEOL ion sputter JFC 1100 instrument. Biofilms were observed using a Schottky Field Emission Scanning Electron Microscope JSM-7900F (JEOL, Tokyo, Japan). Images were obtained at a primary beam energy of 2 kV (SM-EXG65 electron emitter, JEOL, Tokyo, Japan). Regarding experiments involving Saos-2 culture supernatants, one biological experiment for two Saos-2 culture supernatant batches was performed for each condition. Regarding experiments involving primary osteoblast culture supernatants, two biological repeats were performed for each condition.

### 4.8. Confocal Laser Microscopy

For each condition, BS were distributed onto a Thermanox^TM^ coverslip (cell culture treated side up) placed at the bottom of a 24 well plate (500 µL per well). After 24 h of incubation, the coverslips were washed twice in PBS and stained with SYTO^TM^ 9 at 1 µM to label bacteria and (i) propidium iodide (Thermo Fisher Scientific, USA) at 20 µM (in this case to label live bacteria with SYTO^TM^ 9 and damaged or “dead” bacteria with propidium iodide); (ii) SYPRO^®^ Ruby (*v*/*v*) to label proteins; or (iii) wheat germ agglutinin (WGA) associated with the Alexa Fluor^TM^ 350 conjugate at 100 mg/mL to label PIA and TOTO^TM^-3 iodide at 2 mM to label extracellular DNAs (all from Thermo Fisher Scientific, USA). Each label was diluted in 0.9% NaCl. After 30 min of incubation in the dark at room temperature, each coverslip was washed two times with PBS and placed in a 24 well Krystal plate with glass bottom (Porvair, Whiteley, UK) with the biofilm-side downward facing. Acquisitions were performed using CLSM (LSM 710 NLO, Zeiss, Oberkochen, Germany). Fluorochrome-labelled compounds were imaged, and their volume quantified using IMARIS software (v. 9.8.0). Two biological replicates were used for acquisitions. For each coverslip, three representative acquisitions were done.

### 4.9. RT-qPCR (RNA Purification and Reverse Transcription)

For each condition, BS were distributed in six well plates (1 mL per well). After 24 h of incubation, the wells were washed twice in PBS to discard planktonic cells and adherent bacteria were detached with a cell scraper. Total RNAs were extracted from adherent bacteria using MasterPure^TM^ RNA Purification Kit (Lucigen, Middleton, WI, USA) in accordance with the manufacturer’s protocol. Total RNAs were reverse transcribed into complementary DNA (cDNA) using a high-capacity cDNA reverse transcription kit (Applied Biosystems, Sainte Geneviève des Bois, France) following the manufacturer’s instructions. Transcription products were amplified by RT-qPCR using different primers (Eurogentec, Seraing, Belgium) (Table 1) on a StepOne Plus^TM^ system (Applied Biosystems, Villebon-sur-Yvette France). The Takyon^TM^ Rox SYBR^®^ MasterMix (Eurogentec, Seraing, Belgium) was used for amplification. After a first denaturation step at 95 °C for 10 min, RT-qPCR reactions were performed according to a thermal profile that corresponds to 40 cycles of denaturation at 95 °C for 15 s, annealing and extension at 60 °C for 1 min. Data collection was performed at the end of each annealing/extension step. The third step that consists in a dissociation process was performed to ensure the specificity of the amplicons by measuring their melting temperature (Tm). Data analysis was performed with the StepOne^TM^ Software v2.3 (Applied Biosystems, Villebon-sur-Yvette France). Target transcript levels (N-target) were normalized to the housekeeping gene transcript levels, and messenger RNA (mRNA) level with the equation N_target_ = 2^−∆Ct^, where ∆Ct is the Ct value of the target gene after subtraction of Ct for the housekeeping gene. *rho* was used as housekeeping gene. Nine and six biological replicates (three and two per batch) were used for SH1000 and USA300, respectively.

### 4.10. Graphical Representation of Data and Statistical Analysis

Data are presented as histograms (representing the mean; error bars represent standard deviation) or whisker plots (whiskers represent minimum and maximum values, the bottom and top of the box are the 15th and 85th percentiles, and the black band inside the box stands for the median). Each point represents a biological replicate, i.e., the mean of technical replicates (except for CLSM for which technical replicate were considered as individual value). The significance of the results was assessed with an exact non-parametric Wilcoxon–Mann–Whitney test (GraphPad Prism, 8.0.2). When appropriate, stratification was used and then the significance of the results was assessed using StatXact 7.0, Cytel Inc. (Cambridge, MA, USA) and allowed the different Saos-2 culture batches to be taken into account. Differences were considered significant at *p* < 0.05.

## 5. Conclusions

To conclude, we have demonstrated for the first time to our knowledge that osteoblast culture supernatant prepared with human Saos-2 cell line or primary osteoblast have an influence on *S. aureus* biofilm formation, in a strain and donor-dependent manner. Other cells of the bone environment could also disturb biofilm formation. Continued research is warranted to better understand the complex interactions between *S. aureus* and host cells and provide more information about infection establishment. We speculated that SH1000 needed to modify biofilm structure to survive host molecules contrary to the more virulent and resistant USA300 strain which only increased its adherence capacity.

## Figures and Tables

**Table 1:** Nucleotide sequences of primers used for RT-qPCR and efficiency for each primer couple.

Target Gene	Forward Primer (5′ → 3′)	Reverse Primer (3′ → 5′)	Efficiency
*rho*	AACGTGGGGATAAAGTAACTGG	TTCACTTCTTCTGCGTTATGGT	1.9
*icaC*	TCGTATATTTACGTGCCATTATATGTG	AAGCAAGGTGTACCAAAAATGAC	1.9
*sarA*	TTTCTCTTTGTTTTCGCTGATGT	TGTTATCAATGGTCACTTATGCTG	2
*rsh*	CGAAACCTAATAACGTATCAAATGC	TGTATGTAGATCGAAAACCATCACT	2
*cidA*	GATTGTACCGCTAACTTGGGT	GCGTAATTTCGGAAGCAACAT	1.8
*nuc*	TCGAGTTTGACAAAGGCCAA	AAGCAACTTTAGCCAAGCCT	1.9
*fnbpB*	AATTAAATCAGAGCCGCCAGT	AATGGTACCTTCTGCATGACC	2.0

## Data Availability

The data presented in this study are available on request from the corresponding author.

## Supplementary Materials

The supporting information can be downloaded at: https://www.mdpi.com/article/10.3390/ijms232214393/s1.
