# Supplementary material for: Human Osteoblast-Conditioned Media Can Influence Staphylococcus aureus Biofilm Formation
Source: Int J Mol Sci. 2022 Nov 19;23(22):14393. doi: 10.3390/ijms232214393 (PMC9696964; doi:10.3390/ijms232214393)
Supplement: Supplementary file 1 [file ijms-23-14393-s001.zip › ijms-2035043-supplementary.pdf]

International Journal of
Molecular Sciences

MDPI

Supplementary data

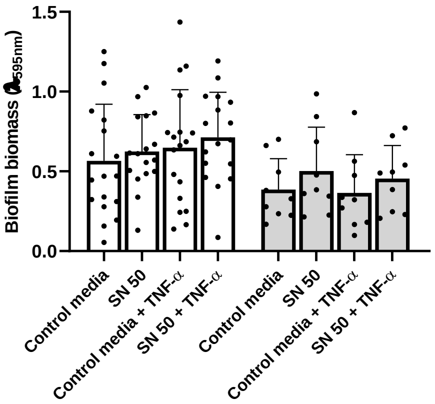

Figure S1. Influence of Saos-2 culture supernatant on *S. aureus* biofilm biomass. Results represent planktonic growth (A), percentage of biofilm biomass relative to Control media (B) and number of adherent bacteria (C) for SH1000 (white histogram) and USA300 (grey histogram). Control media (50 % [DMEM + 10% FBS] and 50 % MM), SN 50 (50 % Saos-2 culture supernatant and 50 % MM), Control media + TNF-*α* (50 % [DMEM + 10% FBS + 20 ng/mL TNF-*α*] and 50 % MM) or SN 50 + TNF-*α* (50 % [Saos-2 challenged with TNF-*α* culture supernatant + 20 ng/mL] and 50 % MM). n = 9 to 17.

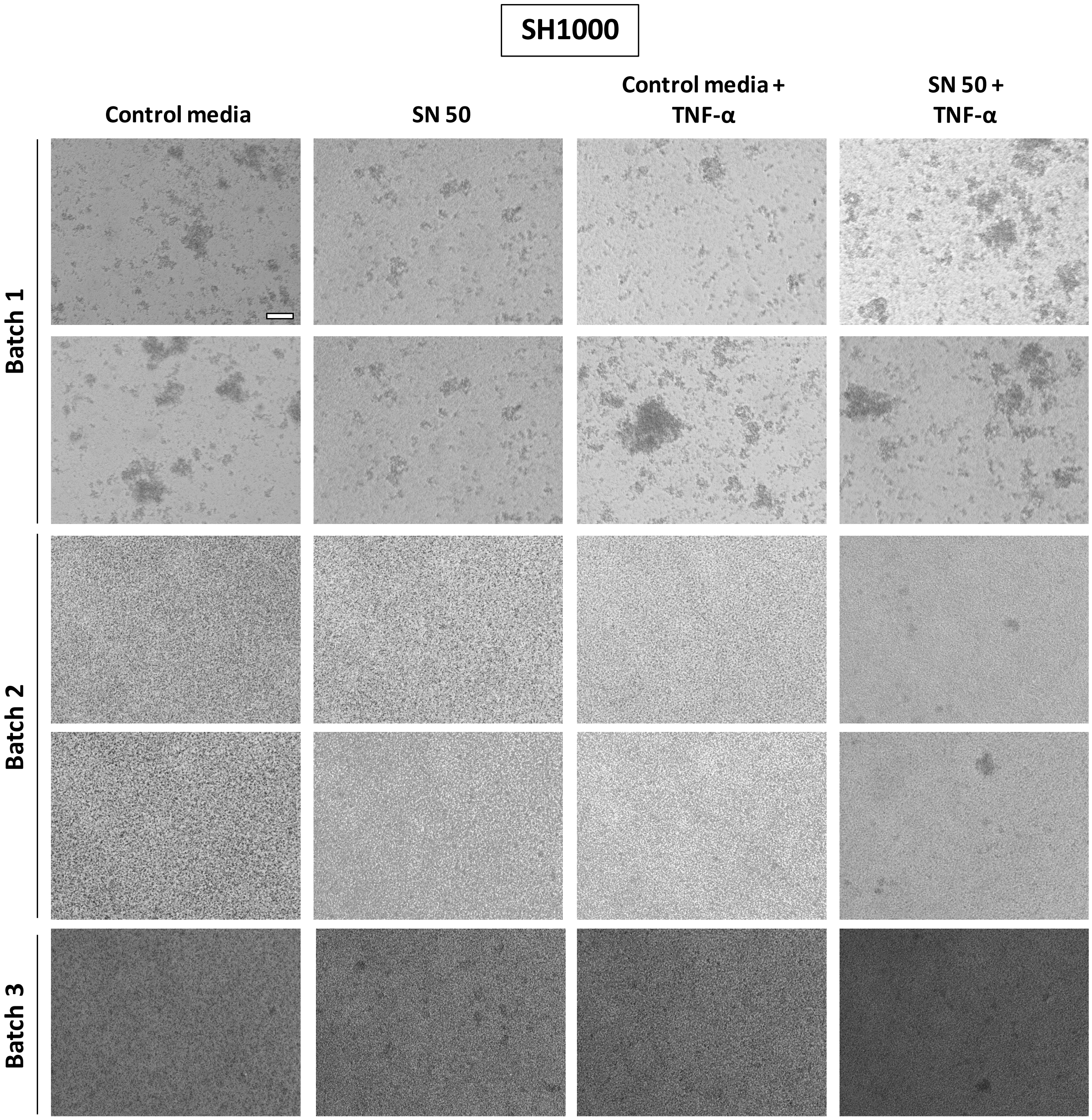
SH1000
Control media
SN 50
Control media + TNF-α
SN 50 + TNF-α
Batch 1
Batch 2
Batch 3

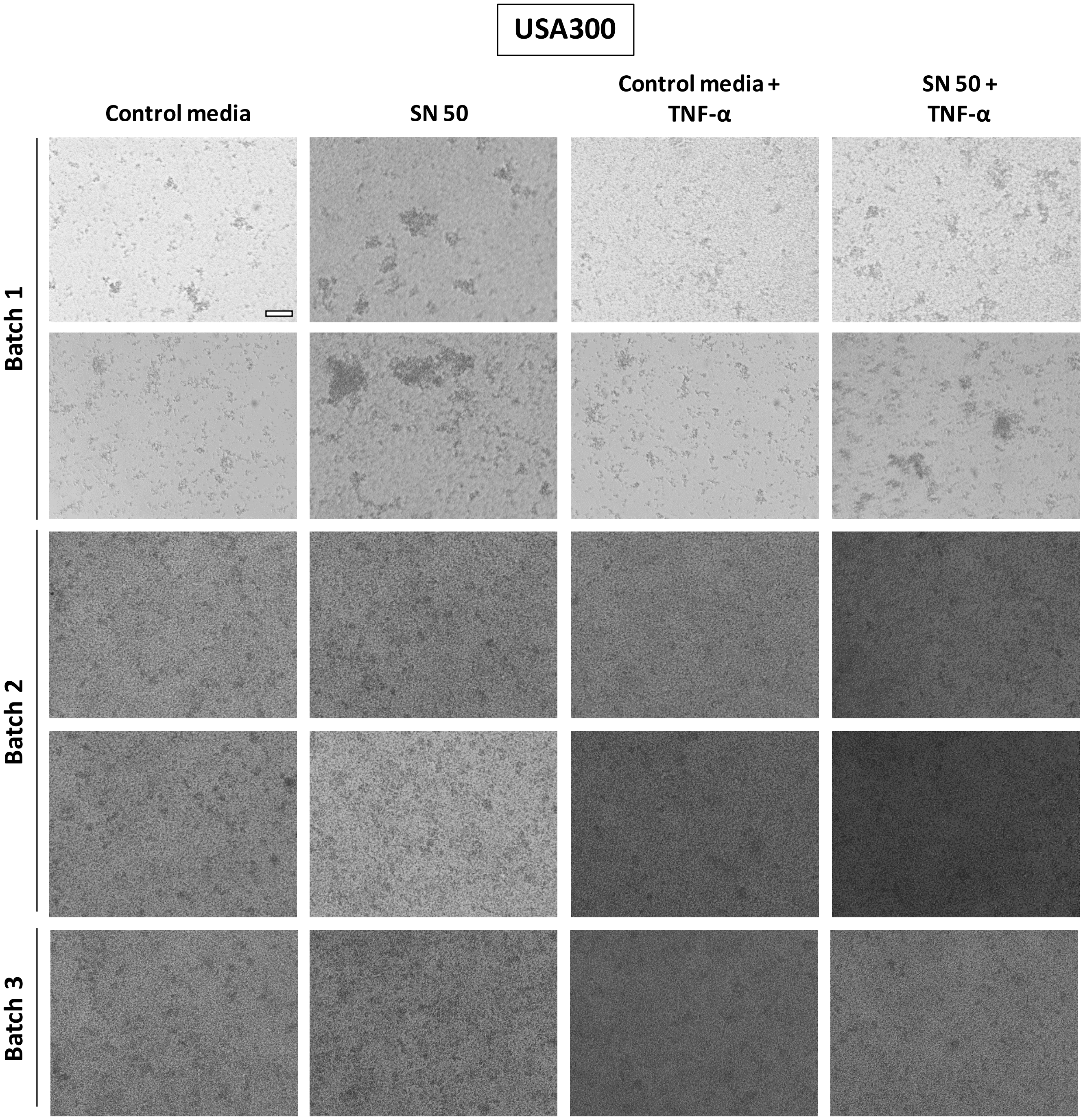

Figure S2. Influence of Saos-2 culture supernatant on *S. aureus* biofilm organization. Other representative images of six experiments (two for three batches) for each condition are shown (corresponding to Figure 2). Control media (50 % [DMEM + 10% FBS] and 50 % MM), SN 50 (50 % Saos-2 culture supernatant and 50 % MM), Control media + TNF-α (50 % [DMEM + 10% FBS + 20 ng/mL TNF-α] and 50 % MM) or SN 50 + TNF-α (50 % [Saos-2 challenged with TNF-α culture supernatant + 20 ng/mL] and 50 % MM). The scale bars indicate 50 μm.

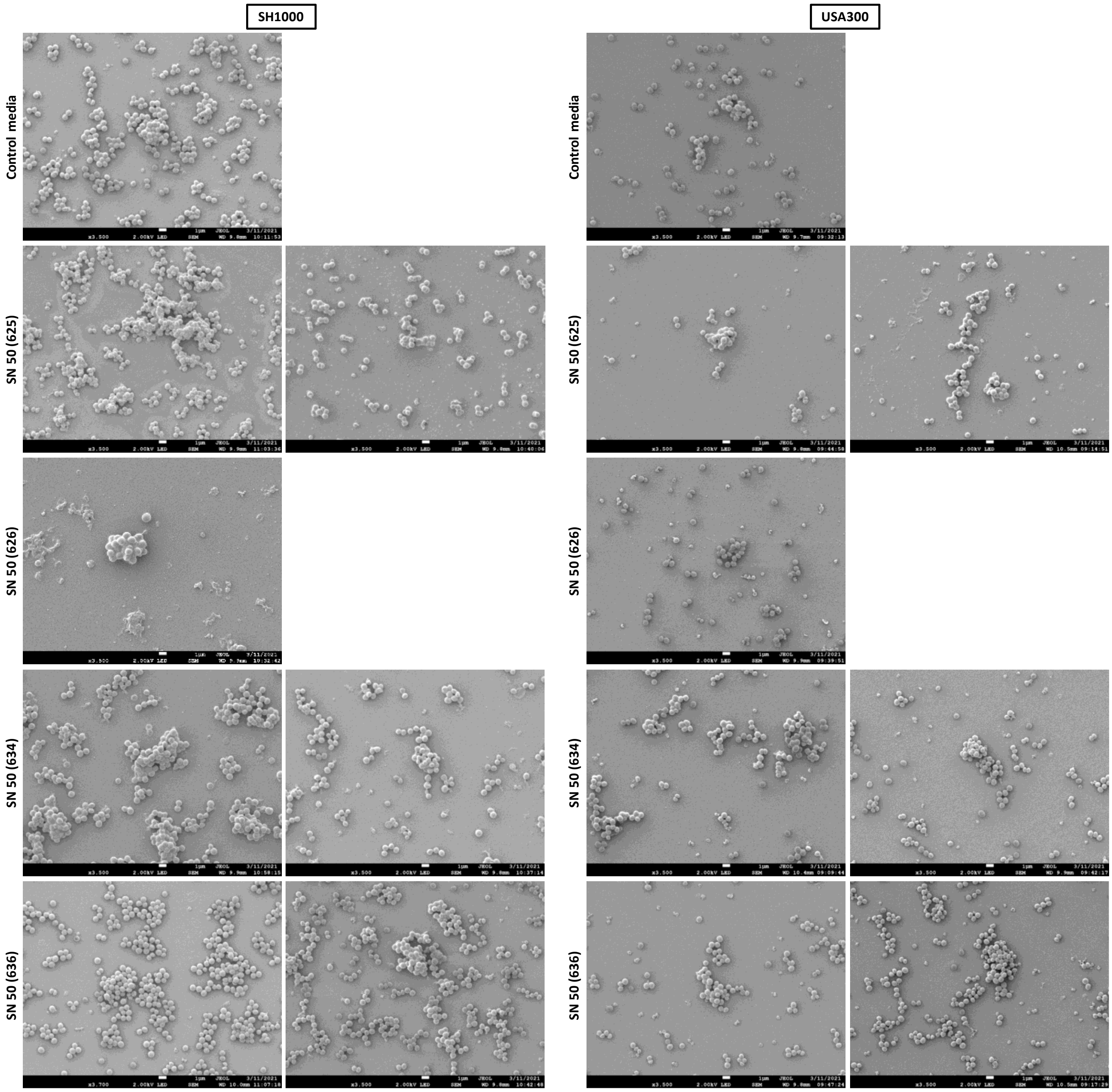

Figure S3. Influence of primary osteoblast culture supernatant on *S. aureus* biofilm. Other representative images of two acquisitions are shown (corresponding to Figure 7). Control media (50 % [DMEM + 10% FBS] and 50 % MM), SN 50 (626) (50 % primary human osteoblast culture supernatant and 50 % MM). The scale bars indicate 1 μm.

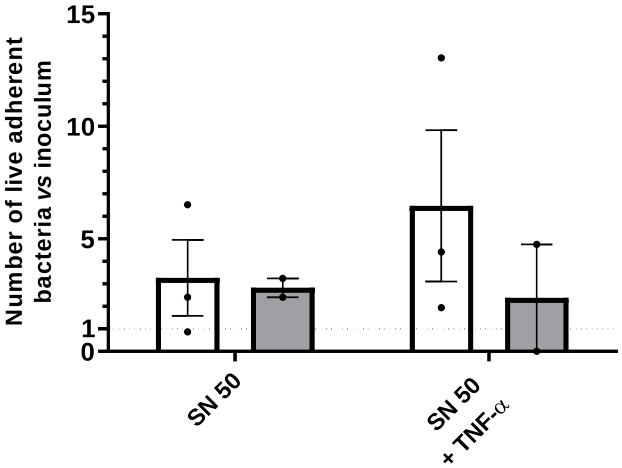

Figure S4. Effect of Saos-2 culture supernatants degraded by proteinase K on SH1000 biofilm growth. Results represent the proportion of adherent and live bacteria versus inoculum. Culture supernatants (SN 50) and culture supernatants from TNF were untreated (white histograms), or treated with proteinase K (100 µg/mL) (Sigma-Aldrich, United States) for 15 min at 65°C and 10 minutes at 90 °C (grey histograms). SN 50 (50 % Saos-2 culture supernatant and 50 % MM); SN 50 + TNF-α (50 % [Saos-2 with TNF-α culture supernatant + 20 ng/mL] and 50 % MM). The experiment protocol corresponds to the Counting method section, except that biofilms were incubated aerobically. n = 2 to 3.

Table S1. Measurement of cytokines contained within Saos-2 and primary osteoblasts culture supernatants. TNF-α, IL-6, IL-8 and MCP-1 were quantified by ELISA (DuoSet®) according to manufacturer's instructions. Results are expressed as mean ± standard deviation (pg/mL). n = 1 to 6.

	**Saos-2 culture supernatants**				**Primary osteoblast culture supernatants**			
	**Control Media**	**SN 50**	**Control Media + TNF-α**	**SN 50 + TNF-α**	**SN50 (625)**	**SN 50 (626)**	**SN 50 (634)**	**SN 50 (636)**
**TNF-α**	0	27.29 ± 39.27	>1000	>1000	0	0	0	0
**IL-6**	0	1 ± 2.07	0	106.2 ± 95.90	>600	314.05	366.55	>600
**IL-8**	0	37.09 ± 21.04	0	>2000	1274.46	176.92	1991.95	>2000
**MCP-1**	0	188.11 ± 176.16	0	>1000	>1000	>1000	>1000	>1000
